# Interreader Reliability of Liver Imaging Reporting and Data System Treatment Response: A Systematic Review and Meta-Analysis

**DOI:** 10.3390/diagnostics11020237

**Published:** 2021-02-04

**Authors:** Dong Wook Kim, Sang Hyun Choi, Ji Sung Lee, So Yeon Kim, So Jung Lee, Jae Ho Byun

**Affiliations:** 1 Department of Radiology and Research Institute of Radiology, University of Ulsan College of Medicine, Asan Medical Center, Seoul 05505, Korea; fsnoruen@gmail.com (D.W.K.); sykim.radiology@gmail.com (S.Y.K.); radiology97@amc.seoul.kr (S.J.L.); jhbyun@amc.seoul.kr (J.H.B.); 2 Department of Clinical Epidemiology and Biostatistics, University of Ulsan College of Medicine, Asan Medical Center, 88 Olympic-Ro 43-Gil, Songpa-Gu, Seoul 05505, Korea; totoro96a@gmail.com

**Keywords:** computed tomography, hepatocellular carcinoma, interreader reliability, LI-RADS, liver, magnetic resonance imaging, meta-analysis, systematic review, treatment response

## Abstract

Background: For a proper management strategy in patients with locoregionally treated hepatocellular carcinoma (HCC), it is essential that the Liver Imaging Reporting and Data System (LI-RADS) treatment response algorithm (LR-TR) has high interreader reliability. We aimed to systematically evaluate the interreader reliability of LR-TR and sources of any study heterogeneity. Methods: Original studies reporting the interreader reliability of LR-TR were identified in MEDLINE and EMBASE up to 20 September 2020. The pooled kappa coefficient (κ) was calculated using the DerSimonian–Laird random effects model. Subgroup analyses were performed according to imaging modality (magnetic resonance imaging (MRI) or computed tomography (CT)). Meta-regression analyses were performed to explore study heterogeneity. Results: Eight studies with 851 HCCs were finally included. Pooled κ was 0.70 (95% CI, 0.58–0.82) for CT/MRI LR-TR, and those of MRI and CT were 0.71 (95% CI, 0.53–0.89) and 0.71 (95% CI, 0.65–0.78), respectively. Study design (*p* < 0.001) and type of treatment (*p* = 0.02) were significantly associated with substantial study heterogeneity. Conclusion: LR-TR showed substantial interreader reliability regardless of the imaging modality. Because of substantial study heterogeneity, which was significantly associated with study design and type of treatment, published values for the interreader reliability of LR-TR should be interpreted with care.

## 1. Introduction

Hepatocellular carcinoma (HCC) is the fifth most common type of cancer and the third leading cause of cancer-related deaths worldwide [1,2]. In the management of HCC, locoregional treatments, including local ablation, transarterial treatment, and external beam radiation therapy (EBRT), play major roles as curative, palliative, or bridging/down-staging therapies [3,4,5,6]. Multiphasic contrast-enhanced computed tomography (CT) and magnetic resonance imaging (MRI) are widely used to evaluate the response after locoregional treatment for HCC. Given the significant correlation between treatment response determined by CT or MRI and patient prognosis [7,8], the accurate and reliable assessment of treatment response by an imaging test cannot be overemphasized.

The Liver Imaging Reporting and Data System (LI-RADS) introduced a treatment response algorithm in 2017 [9]. This provides a comprehensive approach to standardize the assessment of treatment response after locoregional treatments on contrast-enhanced CT or MRI. The LI-RADS treatment response algorithm (LR-TR) evaluates the presence of arterial-phase hyperenhancement, washout appearance, and enhancement similar to that at pre-treatment, and categorizes the treated observation into three categories according to its likelihood of viability, i.e., LR-TR viable, equivocal, or nonviable [9]. 

Notwithstanding the importance of the diagnostic performance, it is essential that the LR-TR has high interreader reliability if it is to be used for determining treatment response and deciding on a proper management strategy in patients with HCC. Prior studies have reported on the interreader reliability of the LR-TR [10,11,12,13,14,15,16,17], but their results are subject to limitations because of their relatively small sample sizes and between-study variability in the reported data. Given the increased attention to LR-TR in clinical practice, we consider it timely and important to determine the interreader reliability of LR-TR and to understand any differences among the published studies.

In this regard, we aimed to systematically evaluate the interreader reliability of LR-TR and explore the sources of any study heterogeneity.

## 2. Materials and Methods

This systematic review and meta-analysis was performed according to the Preferred Reporting Items for Systematic Reviews and Meta-Analyses (PRISMA) guidelines [18].

### 2.1. Literature Search

A systematic search of MEDLINE and EMBASE was conducted to identify original studies reporting the interreader reliability of the CT/MRI LI-RADS treatment response algorithm (LR-TR). The search query is described in Table A1. The bibliographies of the identified studies were explored to search for further eligible studies. As LR-TR was introduced in 2017, the literature search was performed on studies published from 1 January 2017 to 20 September 2020. The search was limited to English-language studies on human subjects.

### 2.2. Eligibility Criteria

Studies that met the following criteria were included: (a) population: patients who underwent locoregional treatment for HCC; (b) index test: multiphasic contrast-enhanced CT and/or MRI; (c) comparator: no comparator; (d) outcomes: interreader reliability of CT/MRI LR-TR. Studies were excluded if they were (a) reviews, conference abstracts, case reports/series, letters, editorials; (b) not in the field of interest; and (c) used a patient cohort that partially overlapped with other studies.

Two reviewers (≥5 years of experience in abdominal imaging) first screened the titles and abstracts for potential eligibility, and then conducted full-text reviews of selected articles to determine their eligibility for the analysis. Disagreements between the reviewers were resolved by consensus.

### 2.3. Data Extraction and Quality Assessment

The following data were extracted from the eligible studies: (a) study characteristics: authors, year of publication, institution, country, duration of patient enrollment, study design (prospective vs. retrospective), and subject enrollment methods (consecutive vs. convenience); (b) patient characteristics: number of patients and HCCs, age, sex, cause of liver disease, locoregional treatment; (c) imaging analysis: imaging modality (CT or MRI), number of readers, and experience of readers; and (d) study outcomes: kappa coefficient (κ) with 95% confidence interval (CI) or standard error.

The quality of the eligible studies was evaluated using the Guidelines for Reporting Reliability and Agreement Studies [19]. Risk of bias regarding the following seven domains was assessed: (a) index test, (b) study subjects, (c) readers, (d) reading process, (e) blinding to reference standard, (f) statistical analysis, and (g) actual numbers of viable/nonviable lesions. Details of the questionnaires regarding each domain are described in Table A2.

The data extraction and quality assessment were independently conducted by the two reviewers, with any disagreements being resolved by consensus.

### 2.4. Statistical Analysis

Using the κ and 95% CI or standard errors reported by the individual eligible studies, the pooled κ with 95% CI for overall LR-TR was calculated using the DerSimonian–Laird random effects model [20]. For the available studies, a subgroup analysis was performed according to imaging modality (CT or MRI). Kappa coefficients of 0–0.2, 0.21–0.4, 0.41–0.6, 0.61–0.80, and 0.81–1 were taken to indicate poor, fair, moderate, substantial, and almost perfect agreement, respectively [21]. Heterogeneity was evaluated using Cochrane’s Q test (*p* < 0.10 indicates substantial heterogeneity) and *I*^2^ statistics (*I*^2^ > 50% indicates substantial heterogeneity) [22]. Publication bias was assessed using a funnel plot and Egger test [23].

To explore the causes of any heterogeneity, meta-regression analyses were performed using the covariates of study design (prospective vs. retrospective), underlying liver disease (hepatitis B dominant vs. others), type of treatment (local ablation vs. transarterial treatment), percentage of LR-TR nonviable category (≥50% vs. <50%), reader number (two vs. three), reader experience (junior (≤5 years of experience)and senior (>5 years of experience) vs. all senior), and statistical methods (unweighted kappa vs. weighted kappa). 

R 4.0.3 (R Foundation for Statistical Computing, Vienna, Austria) was used for statistical analysis.

## 3. Results

### 3.1. Literature Search

**Figure 1:**
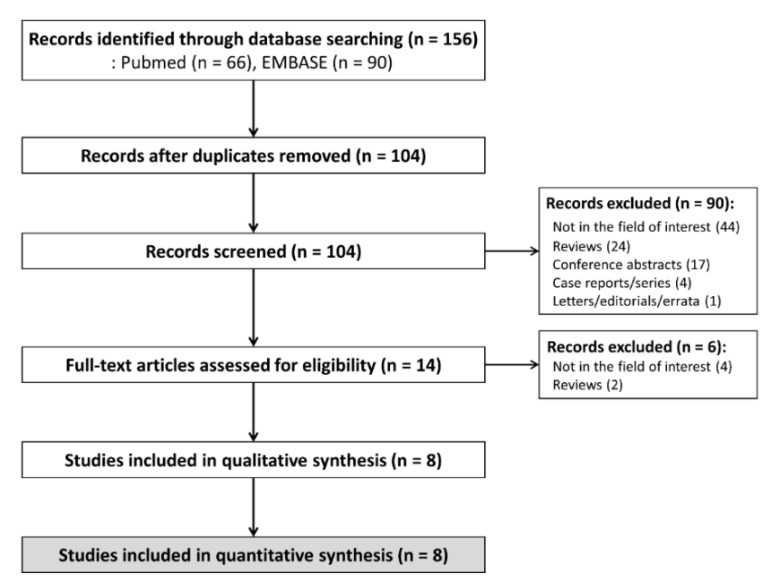
Flow diagram of study selection.

The systematic search identified 156 studies, and 104 were screened after removal of 52 duplicates (Figure 1). After the exclusion of 90 studies by screening of the title and abstract, and six studies by full-text reviews, eight studies were finally included in this systematic review and meta-analysis [10,11,12,13,14,15,16,17]. Because one study [14] investigating the interreader reliability of both CT and MRI had a population overlapping with that of another MRI study [13], the CT interreader reliability result was separately included from this study.

### 3.2. Study Characteristics

The characteristics of the eligible studies are described in Table 1. One [10] collected the patients prospectively whereas the others [11,12,13,14,15,16,17] were retrospective studies. Hepatitis B was the most common underlying liver disease in four studies, which were all from Eastern countries [13,14,15,17]. The population was dominated by patients with hepatitis C and alcoholic hepatitis in three studies from Western countries [11,12,16], while one study did not provide information on underlying liver disease [10]. The locoregional treatment was local ablation only (e.g., radiofrequency ablation, microwave ablation, and percutaneous ethanol ablation) in three studies [11,12,17], transarterial treatment (e.g., conventional transarterial chemoembolization, drug-eluting beads transarterial chemoembolization, and transarterial radioembolization) only in one study [16], and both local ablation and transarterial treatment in four studies [10,13,14,15]. Image analysis was independently performed by two readers in six studies [10,12,13,14,15,17] and by three readers in two studies [11,16]. The readers were all seniors in five studies [10,11,12,15,17], whereas both senior and junior readers performed the analysis in three studies [13,14,16]. 

### 3.3. Quality Assessments

**Figure A1:**
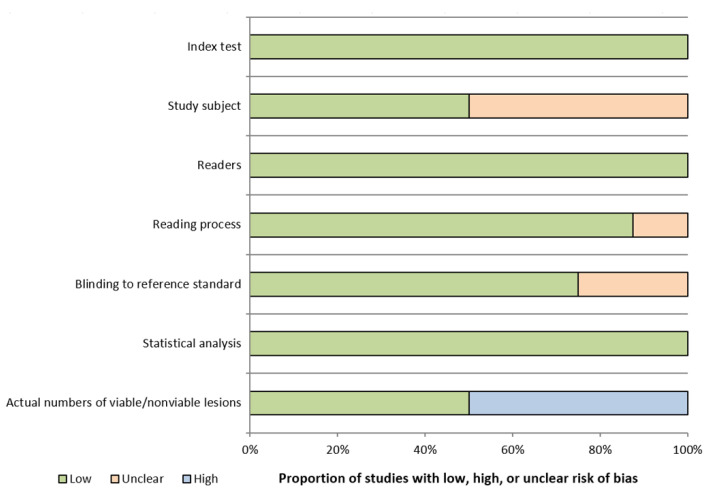
Quality assessment of the eligible studies.

All studies had low risk of bias in more than half of the domains (Figure A1). Four studies [12,13,15,17] had unclear risk of bias regarding the study subjects because it was unclear whether the subjects were consecutively enrolled. Regarding blinding to reference standard, three studies [10,16,17] had unclear risk of bias because they were unclear whether the imaging analysis was blinded to reference standard. Four studies [10,11,12,17] had high risk of bias in the actual numbers of viable/nonviable lesions due to a lack of information regarding viable HCCs. 

### 3.4. Interreader Reliability for LR-TR Category Assignment

**Figure 2:**
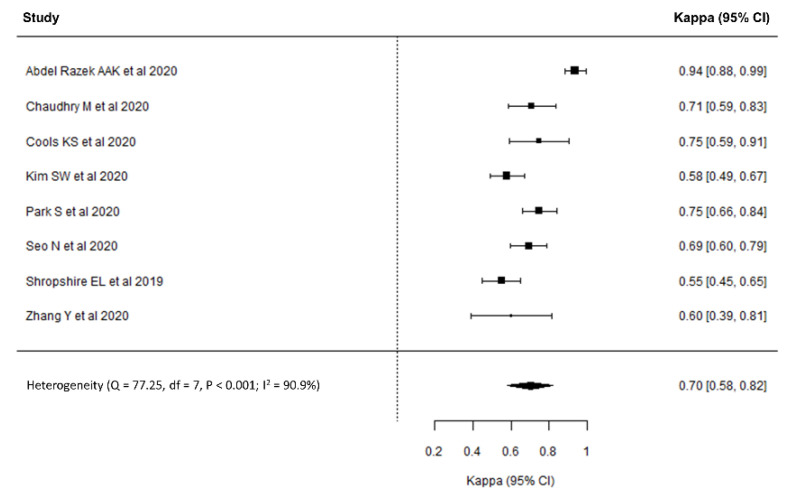
Forest plot of interreader reliability for the Liver Imaging Reporting and Data System treatment response.

**Figure A2:**
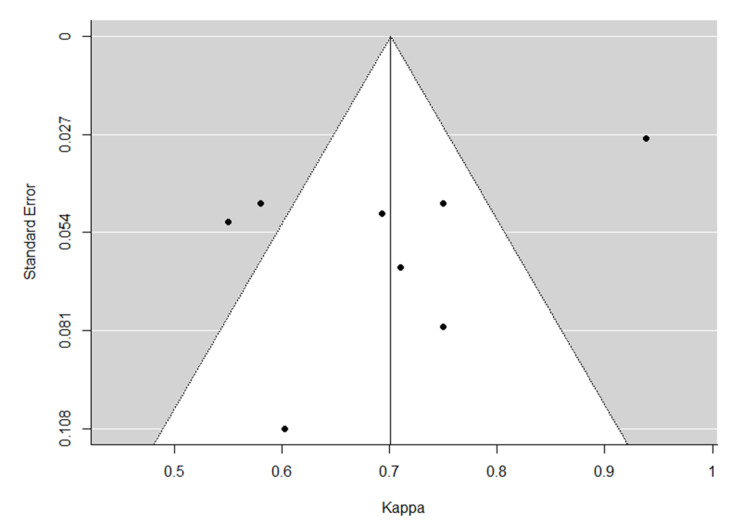
Funnel plot for evaluation of publication bias.

In the eight included studies with 851 HCCs, κ ranged from 0.55 to 0.94, with the pooled κ for LR-TR category assignment being 0.70 (95% CI, 0.58–0.82, Figure 2), indicating substantial interreader reliability. Substantial heterogeneity was observed (Cochrane Q test: *p* < 0.001; I^2^ = 90.9%) but there was no significant publication bias (*p* = 0.33; Figure A2).

**Figure 3:**
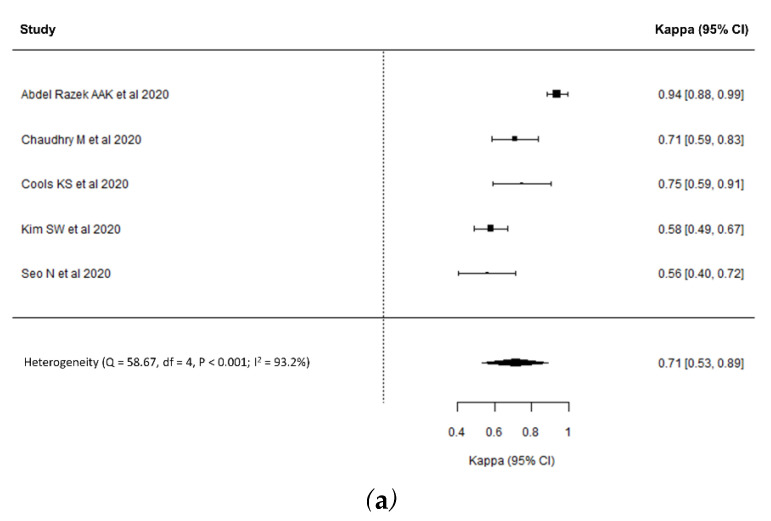

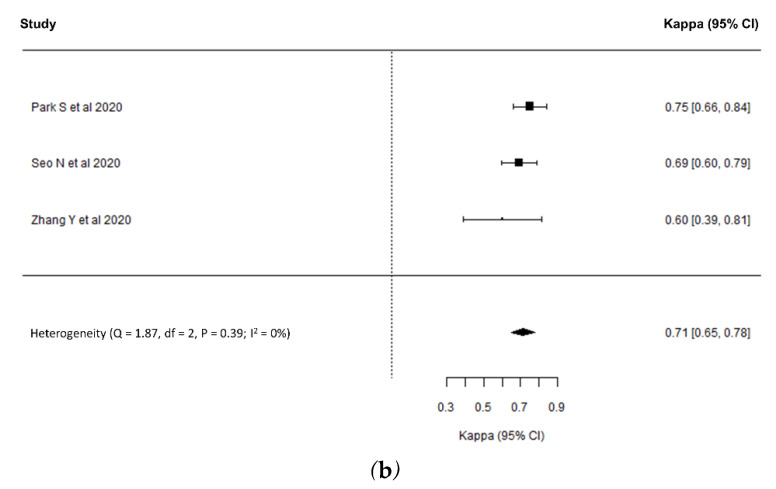
Forest plots of interreader reliability in the subgroups of studies using MRI (**a**) and CT (**b**).

Regarding the imaging modality, the pooled κ values of MRI and CT were 0.71 (95% CI, 0.53–0.89, Figure 3a) and 0.71 (95% CI, 0.65–0.78, Figure 3b), respectively, indicating substantial interreader reliability for both MRI and CT. Substantial heterogeneity was noted in the interreader reliability of MRI (*p* < 0.001; I^2^ = 93.2%) but not in that of CT (*p* = 0.39; I^2^ = 0%).

### 3.5. Meta-Regression Analysis

The results of the meta-regression analyses are described in Table 2. Study design (*p* < 0.001) and type of treatment (*p* = 0.02) were significantly associated with study heterogeneity: prospective studies had a significantly higher pooled κ than retrospective studies (0.94 vs. 0.66), and κ for assessments of LR-TR for HCC after local ablation was significantly higher than that after transarterial treatment (0.70 vs. 0.55). Other covariates were not significantly associated with study heterogeneity.

## 4. Discussion

Our study found that the pooled interreader reliability of LR-TR was substantial (κ, 0.70; 95% CI, 0.58–0.82), and that the interreader reliability of CT was very similar to that of MRI (CT, κ, 0.71 (95% CI, 0.53–0.89) vs. MRI, κ, 0.71 (95% CI, 0.65–0.78)). Study design (prospective vs. retrospective, *p* < 0.001) and the type of treatment (local ablation vs. transarterial treatment, *p* = 0.02) were significant factors affecting study heterogeneity.

LR-TR showed substantial interreader reliability in the assessment of treatment response after locoregional treatment. This interreader reliability of LR-TR was comparable to reported values for other response assessment criteria such as that of the European Association for the Study of the Liver (EASL; κ, 0.69–0.76) and the modified version of the Response Evaluation Criteria in Solid Tumors (mRECIST; κ, 0.67–0.78) [24]. Although LR-TR, EASL, and mRECIST all use arterial-phase hyperenhancement for viability assessment, the major difference in LR-TR is the use of other imaging features including washout and enhancement similar to pre-treatment. Therefore, the interpretation of LR-TR is prone to being more complex and subjective than that of EASL or mRECIST [13,15]. Nevertheless, the strictly standardized definition of viable tumor (i.e., nodular, mass-like, or thick irregular tissue with arterial-phase hyperenhancement, washout, or enhancement similar to pre-treatment) and the reservoir for lesions with indeterminate certainty of viability (i.e., the equivocal category) in LR-TR might explain the comparable results for interreader reliability between LR-TR, EASL, and mRECIST. 

Several previous studies reported conflicting results when determining the optimal imaging modality [25,26,27,28], and in terms of reliability, it is questionable which imaging modality is appropriate for treatment response assessment in HCC after locoregional treatment. According to our study, both CT and MRI showed substantial (κ, 0.71 both in CT and MRI) interreader reliability without a significant difference between them. A plausible explanation for the comparable interreader reliability in this study can be found in the advantages and disadvantages of each imaging modality. For example, in the interpretation of CT after transarterial chemoembolization (TACE), hyperdense lipiodol accumulation precludes accurate assessment by directly obscuring enhancement in the viable portion or by indirectly obscuring it through beam-hardening artifacts [29,30], thus potentially decreasing the reliability of the imaging interpretation. By contrast, iodized oil hardly masks viable HCC on MRI [26,27,31]. However, particularly with the use of gadoxetic acid as a contrast agent, the advantages of MRI can be offset by the weak arterial-phase hyperenhancement due to the relatively small contrast dose and strict washout criteria that are restricted to the portal venous phase according to the current LI-RADS [32]. 

In the meta-regression analysis, the type of treatment was one of the causes of substantial heterogeneity. Although the current LR-TR was designed to assess treated HCC regardless of the type of locoregional treatment, the post-treatment imaging features are specific to each treatment and may therefore cause differences in interreader reliability. Indeed, interreader reliability was significantly higher in local ablation-treated HCC than in TACE-treated HCC (κ, 0.70 vs. 0.55; *p* = 0.02). As we discussed above, hyperdense lipiodol accumulation after TACE may result in uncertainty about whether a residual viable tumor portion is present or not, and may lead to interreader variability in the treatment assessment, particularly when using CT. In addition, tumor heterogeneity caused by partial lipiodol uptake or necrosis in TACE-treated HCC might result in lower interreader reliability than that found for local ablation-treated HCC [33]. Another potential cause of study heterogeneity was the study design. In fact, one prospective study [10] showed significantly higher interreader reliability than the other eligible studies that retrospectively enrolled patients (κ, 0.94 vs. 0.66; *p* < 0.001), and this higher interreader reliability in the prospective study might be explained by the uniform MRI protocol and image analysis [10]. However, our results should be interpreted with caution because the meta-regression analyses were performed using only a small number of studies, numbering eight in total.

Our study has several limitations. First, substantial study heterogeneity was noted, and therefore the single meta-analytic summary estimates may not fully cover the results of the individual studies. To overcome the heterogeneity, we performed a subgroup analysis and meta-regression analyses. Second, locoregional treatment using EBRT could not be evaluated in our study because of a lack of available studies evaluating the interreader reliability of LR-TR EBRT. Considering the slow reduction in size and devascularization after EBRT [34], the interreader reliability of LR-TR after EBRT might differ from that after local ablation or TACE. Third, although our study focused on the interreader reliability of LR-TR, the evaluation of diagnostic performance is also important. Therefore, future study would be necessary to evaluate the diagnostic performance as well as interreader reliability of LR-TR compared with EASL, or mRECIST. 

In conclusion, CT/MRI LR-TR v2017 had substantial interreader reliability regardless of the imaging modality. Substantial study heterogeneity was noted, which was significantly associated with the study design and type of treatment. Because of the presence of substantial study heterogeneity, values for the LR-TR interreader reliability reported in the published literature should be interpreted carefully.

## Figures and Tables

**Table 1:** Study characteristics.

Study	Country	Duration	Study Design	No. of Patients	M:F	Age (Years; Mean ± SD)	Underlying Liver Disease (Number)	No. of HCCs	Treatment	Modality (Contrast Agent [If MRI])	No. of Readers	Reader Experience
Abdel Razek AAK et al. [10]	Egypt	2017.11–2019.01	Prospective	93	79:14	55 ± 2.6	Not available	112	LA (MWA or RFA; 97) TAT (cTACE; 25)	MRI (gadopentetic acid)	2	25 and 10 years in liver imaging
Chaudhry M et al. [11]	USA	2011.01–2015.12	Retrospective	36	32:4	62 ± 5	HCV + alcohol (20) HCV (10) NASH (3) Alcohol (2) HBV (1)	53	LA (MWA or RFA; 53)	MRI (gadobenic acid)	3	8, 7, and 2 years of post-fellowship experience in abdominal MRI
Cools KS et al. [12]	USA	2006.01–2017.12	Retrospective	45	38:7	60 ± 6	HCV (24) HCV + alcohol (13) Alcohol (2) NASH (2) Autoimmune (1)	59	LA (MWA or RFA; 59)	MRI (gadoxetic acid or gadobenic acid)	2	9 years in liver MRI
Kim SW et al. [13]	Korea	2015.01–2016.12	Retrospective	183	133:50	59.9 ± 10.8	HBV (111) Alcohol (34) HCV (25) Others (13)	183	TAT (cTACE or DEB-TACE; 137) LA (RFA; 42) LA + TAT (4)	MRI (gadoxetic acid)	2	7 and 5 years in liver MRI
Park S et al. [14]	Korea	2014.01–2017.12	Retrospective	138	119:19	58 ± 9	HBV (111) HCV (13) Alcohol (6) Others (8)	138	TAT (cTACE, DEB-TACE, or TARE; 98) LA (RFA or PEIT; 18) TAT + LA (22)	CT and MRI (gadoxetic acid)	2	7 and 5 years in liver imaging
Seo N et al. [15]	Korea	2007.01–2014.12	Retrospective	114 (CT, 113; MRI, 53)	96:18	54 ± 6.9	HBV (100) HCV (8) Alcohol (2) Others (4)	206 (CT, 203; MRI, 84)	TAT (cTACE or DEB-TACE; 168) LA (RFA; 34) TAT + LA (4)	CT and/or MRI (gadoxetic acid or gadoteric acid)	2	17 and 16 years in liver MRI
Shropshire EL et al. [16]	USA	2006–2016	Retrospective	45 (CT, 24; MRI, 21)	32:13	57.1 ± 8.2	HCV (22) HCV + alcohol (8) NASH (6) Alcohol (2) AIH (2) NASH + Alcohol (1) HBV (1) Glycogen storage disease (1) Others (2)	63	TAT (cTACE; 63)	CT or MRI (gadoxetic acid or gadobenic acid)	3	17 and 11 years in abdominal MRI
Zhang Y et al. [17]	China	2010.01–2016.12	Retrospective	40	35:5	60.3 ± 10.4	HBV (32) HCV (2) Others (6)	40	LA (RFA; 40)	CT	2	8 and 7 years in liver imaging

CT, computed tomography; cTACE, conventional transarterial chemoembolization; DEB-TACE, drug-eluting beads transarterial chemoembolization: HBV, hepatitis B virus; HCC, hepatocellular carcinoma; HCV, hepatitis C virus; LA, local ablation; MRI, magnetic resonance imaging; MWA, microwave ablation; NASH, non-alcoholic steatohepatitis; PEIT, percutaneous ethanol injection therapy; RFA, radiofrequency ablation; TARE, transarterial radioembolization; TAT, transarterial treatment.

**Table 2:** Meta-regression analyses for interreader reliability in diagnosis using the Liver Imaging Reporting and Data System (LI-RADS) treatment response algorithm (LR-TR)**.**

Covariate	Subgroup	Number of Studies	Estimates	95% CI	*p*-Value
Study design	Prospective	1	0.94	0.88–0.99	<0.001
	Retrospective	7	0.66	0.60–0.73	
Underlying liver disease	HBV dominant	4	0.67	0.58–0.75	0.49
	Others	4	0.74	0.54–0.94	
Type of treatment	Local ablation	3	0.70	0.62–0.79	0.02
	Transarterial treatment	1	0.55	0.45–0.65	
% of LR-TR nonviable	≥50%	5	0.72	0.55–0.90	0.63
	<50%	3	0.67	0.55–0.78	
Reader number	2 (Cohen’s kappa)	6	0.73	0.59–0.86	0.46
	3 (Fleiss kappa)	2	0.63	0.47–0.78	
Reader experience	Junior + Senior	3	0.63	0.50–0.75	0.22
	All senior	5	0.75	0.61–0.89	
Statistical methods	Unweighted kappa	5	0.66	0.58–0.74	0.39
	Weighed Kappa	3	0.76	0.54–0.98	

## Appendix A

**Table A1:** Search queries for MEDLINE and EMBASE.

No.	Search Query for MEDLINE
#1	(((liver neoplasms[MeSH Terms]) OR (Liver[MeSH Terms]))) AND (radiology information systems[MeSH Terms])
#2	(LI-RADS[Text Word]) OR (“liver imaging reporting”[Text Word])
#3	(treatment[Text Word]) OR (response[Text Word])
#4	#1 OR #2
#5	#3 AND #4
#6	LR-TR[Text Word]
#7	#5 OR #6
#8	(((magnetic resonance imaging[MeSH Terms]) OR (“magnetic resonanc*”[Text Word])) OR (MRI[Text Word])) OR (MR[Text Word])
#9	((x ray tomography, computed[MeSH Terms]) OR (“computed tomograph*”[Text Word])) OR (CT[Text Word])
#10	#8 OR #9
#11	#7 AND #10
#12	#11 AND English[Lang]
**No.**	**Search Query for EMBASE**
#1	‘liver imaging reporting and data system’/exp OR ‘liver imaging reporting and data system’
#2	‘li-rads’:ab,ti,kw OR ‘liver imaging reporting’:ab,ti,kw
#3	‘treatment’/exp OR treatment OR ‘response’/exp OR response
#4	#1 OR #2
#5	#3 AND #4
#6	‘lr-tr’
#7	#5 OR #6
#8	‘magnetic resonanc*’:ab,ti,kw OR mri:ab,ti,kw OR mr:ab,ti,kw
#9	‘computed tomograph*’:ab,ti,kw OR ct:ab,ti,kw
#10	#8 OR #9
#11	#7 AND #10
#12	#11 AND [english]/lim

**Table A2:** Questionnaires for quality assessments of the eligible study.

Domain	Questionnaires
Index test	Was information of CT and MRI examination explicitly described?
	Were methods of CT and MRI examination applicable?
Study subject	Was a consecutive or random sample of patients enrolled?
	Was a case-control design avoided?
	Did the study avoid inappropriate exclusion?
Readers	Was information (e.g., number and experiences of readers) explicitly described?
	Were readers representative for general reading practice?
Reading Process	Were readers blinded to clinical information of patients which potentially affected their judgements?
	Was reading process conducted independently?
Blinding to reference standard	Were readers blinded to reference standard which potentially affected their judgements?
Statistical analysis	Was information regarding statistical analysis explicitly described?
	Was statistical analysis applicable?
Actual numbers of viable/nonviable lesions	Was information regarding the actual number of viable/nonviable lesions diagnosed by reference standard described?
